# Do body composition parameters correlate with response to targeted therapy in ER+/HER2- metastatic breast cancer patients? Role of sarcopenia and obesity

**DOI:** 10.3389/fonc.2022.987012

**Published:** 2022-09-23

**Authors:** Endi Kripa, Veronica Rizzo, Francesca Galati, Giuliana Moffa, Federica Cicciarelli, Carlo Catalano, Federica Pediconi

**Affiliations:** Department of Radiological, Oncological and Pathological Sciences, Sapienza University of Rome, Rome, Italy

**Keywords:** body composition, sarcopenia, obesity, metastatic breast cancer, CDK 4/6 inhibitors, automatic segmentation, computed-tomography, visceral adipose tissue

## Abstract

**Purpose:**

To investigate the association between body composition parameters, sarcopenia, obesity and prognosis in patients with metastatic ER+/HER2- breast cancer under therapy with cyclin-dependent kinase (CDK) 4/6 inhibitors.

**Methods:**

92 patients with biopsy-proven metastatic ER+/HER2- breast cancer, treated with CDK 4/6 inhibitors between 2018 and 2021 at our center, were included in this retrospective analysis. Visceral Adipose Tissue (VAT), Subcutaneous Adipose Tissue (SAT) and Skeletal Muscle Index (SMI) were measured before starting therapy with CDK 4/6 inhibitors (Palbociclib, Abemaciclib or Ribociclib). Measurements were performed on a computed tomography-derived abdominal image at third lumbar vertebra (L3) level by an automatic dedicated software (Quantib body composition^®^, Rotterdam, Netherlands). Visceral obesity was defined as a VAT area > 130 cm^2^. Sarcopenia was defined as SMI < 40 cm^2^/m^2^. Changes in breast lesion size were evaluated after 6 months of treatment. Response to therapy was assessed according to RECIST 1.1 criteria. Spearman’s correlation and χ^2^ analyses were performed.

**Results:**

Out of 92 patients, 30 were included in the evaluation. Of the 30 patients (mean age 53 ± 12 years), 7 patients were sarcopenic, 16 were obese, while 7 patients were neither sarcopenic nor obese. Statistical analyses showed that good response to therapy was correlated to higher SMI values (p < 0.001), higher VAT values (p = 0.008) and obesity (p = 0.007); poor response to therapy was correlated to sarcopenia (p < 0.001). Moreover, there was a significant association between sarcopenia and menopause (p = 0.021) and between sarcopenia and the persistence of axillary lymphadenopathies after treatment (p = 0.003), while the disappearance of axillary lymphadenopathies was associated with obesity (p = 0.028).

**Conclusions:**

There is a growing interest in body composition, especially in the field of breast cancer. Our results showed an interesting correlation between sarcopenia and progression of disease, and demonstrated that VAT can positively influence the response to targeted therapy with CDK 4/6 inhibitors. Larger-scale studies are needed to confirm these preliminary results.

**Clinical Relevance:**

Sarcopenia and obesity seem to predict negative outcomes in many oncologic entities. Their prevalence and impact in current breast cancer care are promising but still controversial.

## Introduction

Breast cancer (BC) is the most commonly occurring cancer and the leading cause of cancer death, in women worldwide (1, 2). Nowadays, BC is a complex and heterogeneous disease, which includes several histological subtypes (3).

Therefore, histological and immunohistochemical examination of the biopsy or surgical samples of malignant breast lesions detected on ultrasound, mammography or magnetic resonance imaging (MRI), is fundamental for diagnosis, characterization and treatment choice (4). BC has five recognized molecular subtypes, primarily defined by the presence or the absence of hormone receptors (HR), as summarized in **Table 1** (5).

**Table 1:** Breast cancer molecular subtypes.

Subtype	ER	PR	HER2	Ki-67
Luminal A-like	+	≥ 20%	–	< 20%
Luminal B-like (HER2 negative)	+	-/< 20%^*^	–	≥ 20%^*^
Luminal B-like (HER2 positive)	+		+	
HER2-positive	–	–	+	
Triple-negative	–	–	–	

ER, Estrogen Receptor; PR, Progesterone Receptor.

*Only one of these criteria must be met.

To date, BC therapy is decided in a multidisciplinary setting. The Eighth Edition of the AJCC classification, currently in use, takes into account the anatomical extension of BC, in terms of local and systemic extent of disease, and a prognostic classification (Prognostic Stage Group) which includes tumor grade, HR status, and HER2 status. Therapy involves the combination of neoadjuvant therapy (in locally advanced and inoperable BC), surgery, radiotherapy and adjuvant chemotherapy and/or endocrine therapy (6–9).

There has been an important evolution in therapeutic strategies through the years, due to the identification of additional prognostic and predictive factors. Among these, recently body composition parameters, in particular muscle mass and adipose tissue distribution, have been identified as interesting prognostic markers (10). There is a growing interest in the role of body composition in BC management, as it has been suggested that it can influence the response to therapy and the progression of disease (11, 12).

The aim of our study was to evaluate whether sarcopenia and obesity could predict the response to therapy in patients with metastatic ER+/HER2- BC, treated with CDK 4/6 inhibitors.

The term sarcopenia indicates a reduction in muscle strength and mass (13). Obesity is defined as a body mass index (BMI) of ≥ 30 kg/m^2^ (14). However, several studies have shown that BMI alone is not a sufficient and accurate parameter to assess obesity and to evaluate outcomes and prognosis in cancer patients, especially when compared with the degree of visceral obesity (15–17), since BMI lacks in distinguishing between fat and lean mass and between visceral and subcutaneous adipose tissue. As known, visceral fat is more metabolically active than subcutaneous one, and leads to chronic inflammation and tumorigenesis (18).

The ER+/HER2− BC subtype is commonly treated using hormone-based therapies in the adjuvant setting, with significant survival benefit associated with these therapies even in the metastatic setting (19). However, hormone resistance develops in most metastatic patients (20, 21). For this reason, the current standard of care for most patients with ER-positive metastatic BC consists in CDK 4/6 inhibitors as first-line therapy, in association with aromatase inhibitors or combined with Fulvestrant, as second-line therapy (22, 23). CDK 4/6 are cell cycle regulators that control the rate of growth and division of cells and check important metabolic processes (24, 25). In metastatic BC, these proteins can become overactive, causing uncontrollable cell growth and division. CDK 4/6 inhibitors suppress CDK 4/6 proteins, blocking the transition from the G1 to the S phase of the cell cycle, in order to slow down or even to stop cancer cells replication. Furthermore, recent preclinical studies have identified potential targets for diet-induced obesity in CDK 4 and 6, suggesting that the use of CDK 4/6 inhibitors could have a direct effect on body fat mass and muscle mass (26, 27). Finally, in recent years, muscle and adipose tissue measurements have received increasing attention as potential prognostic factors as well as predictors of treatment-related toxicity (28, 29). Thus, the ultimate aim of our research was to investigate if the amount of adipose tissue, skeletal muscle mass, obesity and sarcopenia are predictive of a positive or negative prognosis in metastatic ER+/HER2- BC patients.

## Materials and methods

### Study design

Our research is a retrospective study on patients with a diagnosis of metastatic ER+/HER2- BC, treated with CDK 4/6 inhibitors.

The study was performed according to the Declaration of Helsinki and approved by the Institutional ethics committee. Written informed consent was waived because of the retrospective design of this study.

Between November 2018 and November 2021, 92 patients with metastatic ER+/HER- BC treated with CDK 4/6 inhibitors combined with endocrine therapy were included in the analysis.

Inclusion criteria were: female gender, age between 18 and 85 years, biopsy-proven (ultrasound-guided or stereotactic) metastatic ER+/HER2- invasive BC, availability of computed tomography (CT) images and breast MRI examination at baseline and after 6 months of treatment.

Patients unsuitable for treatment with CDK 4/6 inhibitors, previously treated with tamoxifen, aromatase inhibitors or neoadjuvant chemotherapy (within 30 days prior to enrollment), previously treated with radiotherapy or ablative therapy of the affected breast, pregnant, breastfeeding or in postnatal period, and patients who had any contraindication to perform CT and/or MRI examinations, were excluded.

### Staging

Staging multiphasic CT was performed by using a multidetector CT scanner (Somatom Sensation 64; Siemens Healthineers, Erlangen, Germany). CT scan was performed using a fixed tube voltage of 120 kVp, with automatic exposure control, and an image slice thickness of 1–5 mm. CT scan protocol included a non contrast phase, followed by a late arterial phase and a portal venous phase acquisition.

**Figure 1:**
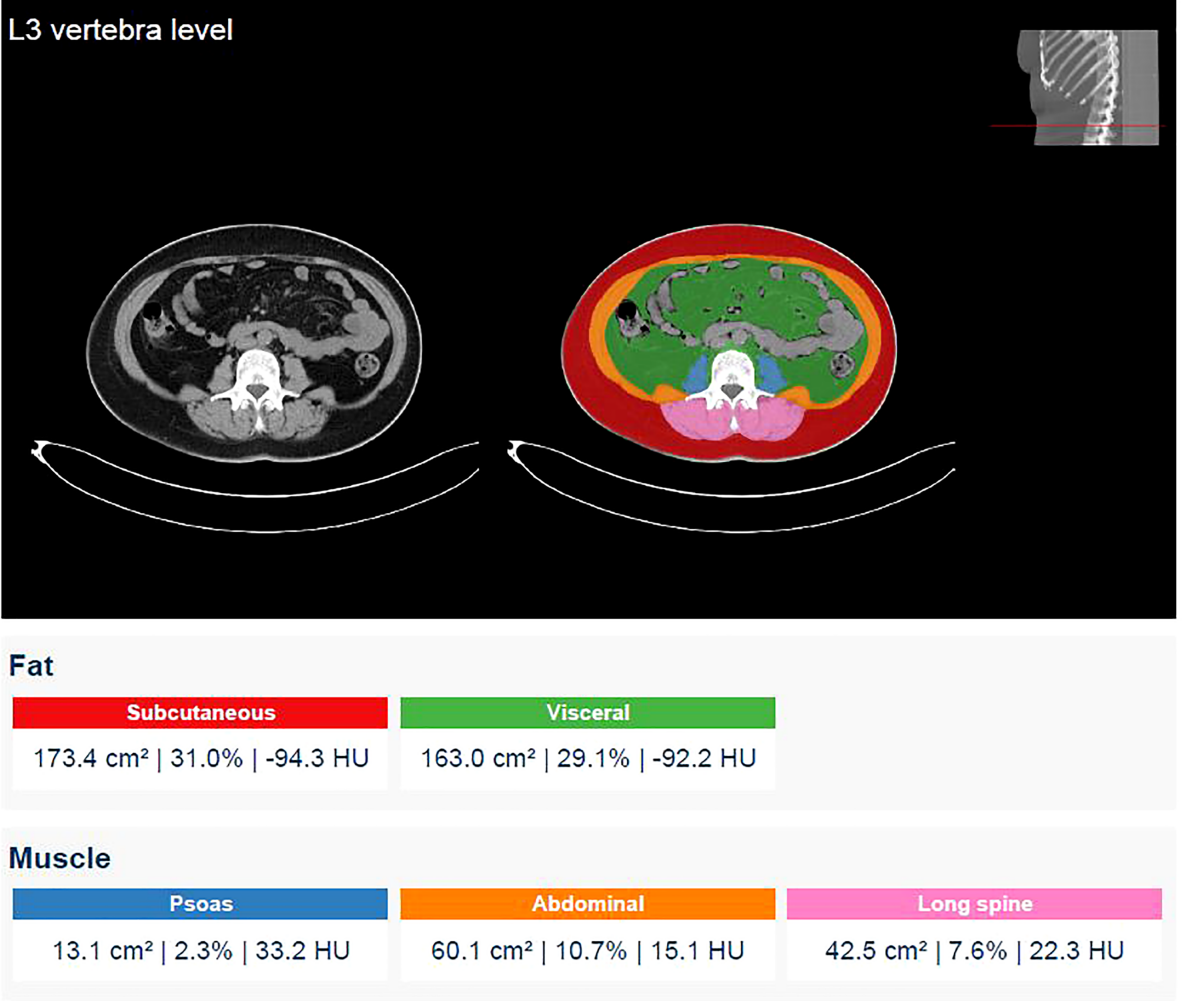
Screenshot generated by the software after performing an automatic segmentation at the level of the third lumbar vertebra and report of the measurements of SAT, VAT and SMA carried out. SAT values are indicated in red and VAT values in green. SMA is obtained by summing the areas of the psoas, the abdominal and the long spinal muscles indicated in blue, orange and pink respectively (13.1 + 60.1 + 42.5 = 115.7 cm^2^). SMI is then calculated as the ratio of SMA to the height squared of the patients.

Body composition was quantitatively assessed using an automatic segmentation software named Quantib body composition^®^ (Rotterdam, Netherlands) (30, 31). Quantib^®^ evaluated the patient CT-derived image extracted from staging CT examinations (non-contrast or portal venous phase series) in our institutional picture archiving and communication system (PACS). The software performs an automatic segmentation at the level of L3 vertebral body to calculate subcutaneous adipose tissue (SAT), visceral adipose tissue (VAT), and skeletal muscle area (SMA). Lastly, the software automatically generates a form containing the values of interest, as in **Figure 1**.

Skeletal Muscle Index (SMI) was then calculated as the ratio of SMA to the patient’s squared height. According to literature, a VAT area > 130 cm^2^ indicates obesity and a SMI value < 40 cm^2^/m^2^ is considered a cut-off value for sarcopenia (32, 33). Therefore, patients were divided into normal weighted, obese, non-sarcopenic and sarcopenic, accordingly.

All patients also underwent a first MRI examination at baseline, for loco-regional staging, and at least a second MRI examination 6 months after the beginning of targeted therapy, to evaluate the change in size of the target lesion during therapy. All breast MRI were performed on a 3T magnet (Discovery MR 750; GE Healthcare, Chicago, IL, USA) with a dedicated 8-channel breast coil compatible with parallel imaging, and patients in a prone position. Breast MRI protocol included: axial pre-contrast 2D FSE T2-weighted fat-suppressed sequence (repetition time [RT] = 9000–11,000 ms, echo time [ET] 119–120 ms, matrix = 512 × 224, slice thickness = 3–5 mm, field of view [FOV] = 35 × 35 cm, NEX = 1, scan time = 130 s), axial pre-contrast diffusion-weighted echo-planar imaging (DWI-EPI) sequence (RT = 4983–5314 ms, ET = 58 ms, matrix = 150 × 150, slice thickness = 3–5 mm, FOV = 350 × 350 mm, NEX = 2–2–4, scan time = 230 s), axial dynamic three-dimensional (3D) spoiled gradient-echo T1-weighted fat-suppressed sequences (flip angle = 15°, RT = 8 ms, ET = 4 ms, matrix = 512 × 256, slice thickness = 1.40 mm, FOV = 380 × 380 mm, NEX = 1) and sagittal 3D spoiled gradient-echo post-contrast T1-weighted sequence.

### Follow-up

After 6 months of therapy, a re-stadiation CT and MRI were performed to evaluate distant metastases and local disease, respectively.

RECIST 1.1 criteria (34) were used to identify and classify the response to therapy. A Complete Response (CR) was defined by the disappearance of all target lesions and the reduction in short axis to < 10 mm of any pathological lymph nodes; Partial Response (PR), in case of at least a 30% decrease in the sum of diameters of target lesions; Progressive Disease (PD), in case of at least a 20% increase in the sum of diameters of target lesions and an absolute increase of at least 5 mm; Stable Disease (SD), in all other cases.

After 6 months of CDK 4/6 inhibitors therapy, we considered CR and PR as good response to therapy, while PD and SD as poor response.

### Statistical analysis

Statistical analysis was performed using IBM^®^ SPSS Statistics, version 25. The Kolmogorov-Smirnov Z test was performed to assess the normality of the distribution for all continuous variables. A Spearman’s analysis was carried out to verify the correlation between variables. A comparison of categorical variables was performed using the χ^2^ test.

P-values < 0.05 were considered statistically significant.

## Results

### Study population

From the 92 patients included in the analysis, 6 patients discontinued therapy due to the onset of toxic side effects; 14 patients could not perform or complete CT or MRI examinations because of allergy to contrast media or claustrophobia; 33 patients had incomplete CT or MRI imaging sets; 8 patients waived the follow-up; 1 patient died for other causes. Thus, 30 patients were suitable for the evaluation.

Of the 30 patients (mean age of 53 ± 12 years) with metastatic ER+/HER2- BC included in the study and treated with CDK 4/6 inhibitors, 19 patients (63.3%) were in menopause while 11 (36.7%) were not. Obesity was present in 16 patients (53.3%), normal weight in 14 (46.7%) and sarcopenia in 7 patients (23.3%). None of the patients were both sarcopenic and obese.

All patients had axillary lymph node metastases at baseline local staging MRI and CT. For what concerns distant metastases, 12 patients (40.0%) had liver metastases, 13 patients (43.3%) had skeletal metastases, 1 (3.3%) patient had lung metastases, and 4 patients (13.3%) had both bone and brain metastases. There were no differences regarding sites of metastases between sarcopenic and non-sarcopenic patients and between obese and normal weighted patients.

### Correlation of baseline body composition parameters and response to therapy

After 6 months of therapy, 4 patients (13.3%) had PD. All 4 patients had an increase in target lesion’s size of at least 20% and 2 of them had new metastases on 6-month follow-up CT scan. 4 patients showed SD (13.3%). 22 (73.3%) patients had CR or PR to treatment.

The Kolmogorov-Smirnov Z test demonstrated a non-normal distribution in the lesions size, both before and after therapy; the test showed a normal distribution of the variables SAT, VAT, SMA and SMI.

The median of lesions size assessed before and after chemotherapy is respectively 25,5 cm (5 - 100 cm) and 13,5 cm (0 - 58 cm).

At CT-scan analysis, BC patients showed a mean VAT of 104.8 cm^2^ (SD 62.39), SAT of 213 cm^2^ (SD 89.73), SMA of 128 cm^2^ (SD 33.51) and SMI of 49.75 cm^2^/m^2^ (SD 13.54).

Spearman’s analysis demonstrated a statistically significant correlation between higher VAT values and a good response to therapy (p = 0.008) and also between higher SMI values and a good response to therapy (p < 0.001). Furthermore, a direct correlation was found between VAT and SMI values (p = 0.04). Further details are included in **Table 2**.

**Table 2:** Statistical correlation between response to therapy and body composition parameters.

Spearman's Rho		Response to therapy	SAT	VAT	SMA	SMI L3
Response to therapy	Correlation coefficient	1.000	0.100	**0.476**	**0.643**	**0.687**
	*p-value*		0.600	**0.008**	**< 0.001**	**< 0.001**
SAT	Correlation coefficient	0.100	1.000	**0.507**	0.051	0.049
	*p-value*	0.600		**0.004**	0.787	0.799
VAT	Correlation coefficient	**0.476**	**0.507**	1.000	**0.413**	**0.376**
	*p-value*	**0.008**	**0.004**		**0.023**	**0.040**
SMA	Correlation coefficient	**0.643**	0.051	**0.413**	1.000	**0.937**
	*p-value*	**< 0.001**	0.787	**0.023**		**< 0.001**
SMI	Correlation coefficient	**0.687**	0.049	**0.376**	**0.937**	1.000
	*p-value*	**< 0.001**	0.799	**0.040**	**< 0.001**	

Statistically significant results are bolded (p-value <0.05).

χ^2^ analysis showed a statistically significant association between sarcopenia and the persistence (no detectable modifications in terms of size and morphology at MRI) of axillary lymphadenopathies after therapy (p = 0.003), between sarcopenia and menopause (p = 0.021) and between sarcopenia and a worse response to therapy (p < 0.001). χ^2^ also found that obesity was associated with a good response to therapy (p = 0.007) and with the absence of axillary lymphadenopathies after therapy (p = 0.028). No significant association was found between obesity and menopause (**Table 3**).

**Table 3:** Association between sarcopenia and obesity and clinical parameters.

χ^2^ test	Sarcopenia (7/30)	Obesity (16/30)
Menopause		
Yes	7/7	10/16
No	0/7	6/16
* p-value*	**0.021**	0.92
Response to therapy		
Good response	0/7	15/16
Poor response	7/7	1/16
* p-value*	**<0.001**	**0.007**
Lymphadenopathies after treatment		
Presence	7/7	5/16
Absence	0/7	11/16
* p-value*	**0.003**	**0.028**

Statistically significant results are bolded (p-value <0.05).

## Discussion

In recent years, there is a growing interest in the role of body composition parameters, sarcopenia and obesity status in several oncologic entities, including BC, colorectal cancer (35), prostate cancer (36) and leukemia (37).

It has been established that BMI cannot be sufficiently accurate as a stand-alone parameter to assess body composition (10, 33). An alternative method commonly used to quantify body composition is dual X-ray absorptiometry (DEXA), characterized by a relatively low radiation exposure, low costs and the ability to evaluate the whole body in a single scan. A further imaging modality in addition to DEXA is CT, that is able to provide details on specific muscles, visceral and subcutaneous adipose tissue while performing a whole body staging or follow-up in cancer patients. Moreover, CT combines clinical accessibility with high accuracy in the quantification of specific tissues and in the evaluation of body composition (38).

In patients with metastatic BC undergoing endocrine therapy alone, the relationship between BMI and response to therapy is still controversial. In particular, Zewenghiel et al. have described no differences in treatment efficacy between normal, overweight and obese patients with metastatic BC treated with Fulvestrant (39). On the contrary, Gevorgyan has reported a worse response in obese patients treated with Fulvestrant (40).

**Figure 2:**
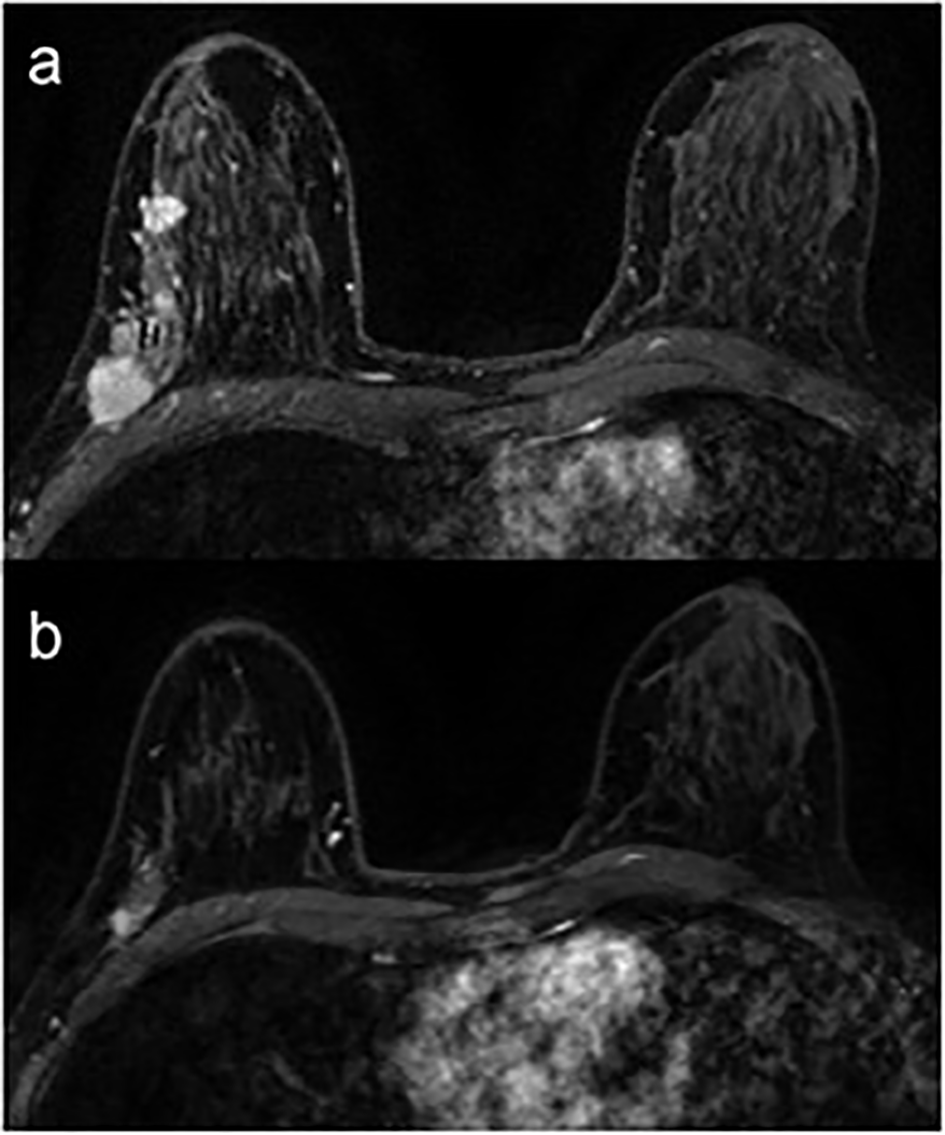
Breast MRI of a 53-year-old obese woman (VAT = 152.8 cm^2^; VAT > 130 cm^2^ = visceral obesity) with a multifocal breast cancer of the right breast. Axial post-contrast T1-weighted images before **(A)** and after **(B)** 6 months of CDK 4/6 inhibitors show a good response to treatment, as documented by the reduction in size of all lesions in the upper-outer quadrant/axillary extension of the right breast.

Focusing on VAT, in agreement with Franzoi et al. (40), our results have shown a correlation between the absolute value of VAT and the response to therapy with CDK 4/6 inhibitors. This indicates thata higher VAT value, and therefore the state of obesity, can predict a better response to treatment (**Figure 2**). We supposed that better outcomes of our patients with higher VAT could be due to the increased expression of CDK 4/6, since CDK 4/6 play an important role in adipogenesis (24). It has been demonstrated that CDK 4 influences adipocyte differentiation and function through the activation of peroxisome proliferator-activated receptor gamma (PPARγ) and that the disruption of CDK 4 or the presence of activating mutations in CDK 4 in primary mouse embryonic fibroblasts result in reduced and increased adipogenic potential of these cells, respectively (41). Nevertheless, Pizzuti et al. have shown a negative impact of obesity in terms of Progression Free Survival (PFS) in patients with endocrine resistant metastatic BC treated with Fulvestrant (42). Their results, apparently in contrast with ours, could depend on the different therapeutic line studied or on the assessment of the state of obesity based on BMI and not on VAT values. On the contrary, Franzoi et al. (43) have shown that patients with high visceral fat index had a longer PFS compared to patients with low visceral fat index.

Regarding low muscle mass and response to treatment, recently many authors have investigated the role of sarcopenia in several oncologic entities, included BC, both in terms of response to therapy and in relation to toxic side effects associated with chemotherapy, suggesting that low muscle mass in cancer patients is an important prognostic factor in terms of treatment-induced toxicity and survival (44, 45).

**Figure 3:**
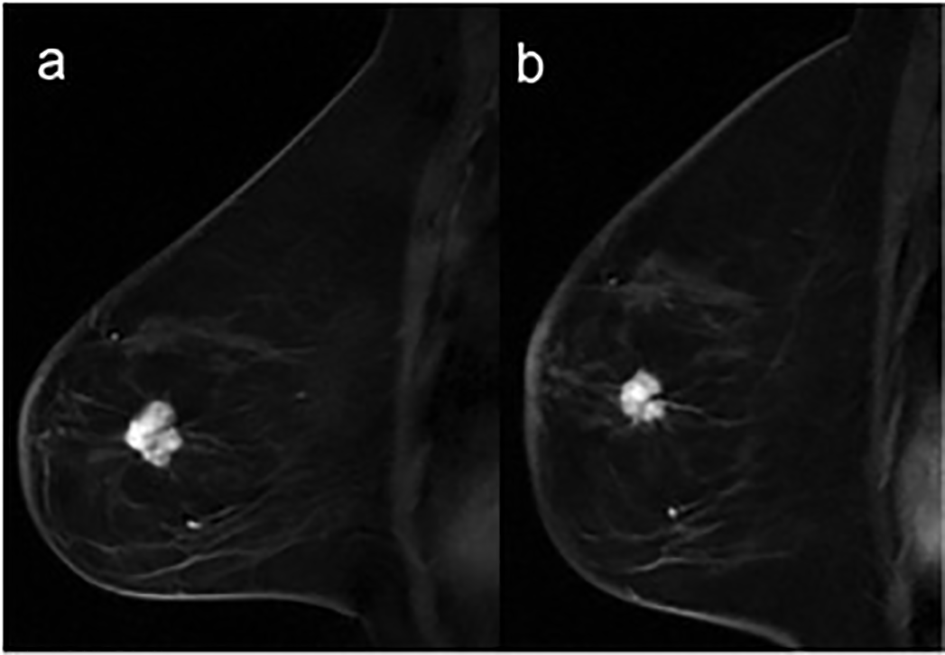
Breast MRI of a 57-year-old sarcopenic woman (SMI = 34.3 cm^2^/m^2^; SMI < 40 cm^2^/m^2^ = sarcopenia) with invasive ductal carcinoma of the left breast. Sagittal post-contrast T1-weighted images, before **(A)** and after **(B)** 6 months of treatment shows a substantial stability in the size of the lesion located in the lower-outer quadrant of the left breast.

Our study has shown that sarcopenia could be a negative prognostic factor in patients with metastatic ER+/HER2- BC treated with CDK 4/6 inhibitors (**Figure 3**), in accordance with the aforementioned recent study by Franzoi (43). Our analysis has also confirmed that CT is an important diagnostic tool in the evaluation of sarcopenia and adiposity, in agreement with a previous study by Cruz-Jentoft (46). Caan et al. also measured sarcopenia by CT among 3241 patients and has reported an increased risk of death in patients with early BC presenting this condition (33). On the contrary, Rier et al. (47) have shown that low muscle density was associated with worse overall survival (OS) or PFS in metastatic BC patients treated with 1st-line FAC (Fluorouracil, Doxorubicin and Cyclophosphamide) or Paclitaxel, unlike sarcopenia. Prado et al. have reported sarcopenia as a determinant factor for higher chemotherapy-related toxicity and shorter time to tumor progression in 55 metastatic BC patients treated with Capecitabine after Taxane and/or Anthracycline progression (28).

A further notable finding was the significant correlation between sarcopenia and menopause. This could be explained by the drop in estradiol levels in menopause. As demonstrated by Geraci et al. (48), estradiol is an important factor related to the development of sarcopenia, as it can promote muscle regeneration, contributing to its health, and is also involved in the modulation of the local and systemic inflammatory responses (49, 50).

Statistical analysis revealed a direct correlation between VAT and SMI values. To our knowledge, this finding is not supported by other studies in the literature. The result could be occasional and without clinical relevance. Further studies with a larger population could confirm or reject our conclusion.

Our study has some limitations. It is a retrospective, monocentric analysis with a limited number of patients. Even if our work concerns ER+/HER2- BC treated with CDK 4/6 inhibitors, it would be interesting to evaluate how body composition affects other BC subtypes and therapeutic lines.

The main originality of our study is due to the dedicated software we used (Quantib body composition^®^), which automatically segments VAT, SAT and SMA on CT images. The use of a full-automated segmentation software presents numerous advantages. First, the lack of bias for manual segmentation, such as the visual determination of the different anatomical compartments. Furthermore, the automatic detection of the soma of L3 avoids centering errors. Finally, the image analysis time is drastically reduced. Another strength of our study is the local staging performed with breast MRI, which is the most sensitive imaging modality in terms of BC detection and assessment of tumor size (51), providing accurate information on tumor extent, skin and nipple invasion, and nodal involvement.

## Conclusions

Our study confirms and corroborates the role of sarcopenia as a potential early predictor of poor prognosis in metastatic BC patients treated with CDK 4/6 inhibitors. Furthermore, this study highlights the value of VAT measurement as a more accurate indicator of obesity than BMI and demonstrates that an increase in VAT could be associated with a better prognosis in metastatic ER+/HER2- BC patients. Further large-scale studies are needed to validate the predictive role of sarcopenia and VAT during CDK 4/6 inhibition.

## Data availability statement

The raw data supporting the conclusions of this article will be made available by the authors, without undue reservation.

## Author contributions

Conceptualization: EK, VR and FG; Material preparation, data collection and analysis: EK, VR, FC. Writing: EK, GM. Supervision: VR, GM, and FG. Validation: FG, CC, FP. All authors contributed to the article and approved the submitted version.

## Conflict of interest

The authors declare that the research was conducted in the absence of any commercial or financial relationships that could be construed as a potential conflict of interest.

## Publisher’s note

All claims expressed in this article are solely those of the authors and do not necessarily represent those of their affiliated organizations, or those of the publisher, the editors and the reviewers. Any product that may be evaluated in this article, or claim that may be made by its manufacturer, is not guaranteed or endorsed by the publisher.
